# Prevalence of Epilepsy in Frontotemporal Dementia and Timing of Dementia Diagnosis

**DOI:** 10.1001/jamaneurol.2025.1358

**Published:** 2025-06-02

**Authors:** Annemari Kilpeläinen, Mikko Aaltonen, Kalle Aho, Sami Heikkinen, Ave Kivisild, Adolfina Lehtonen, Laura Leppänen, Iina Rinnankoski, Helmi Soppela, Laura Tervonen, Päivi Hartikainen, Annakaisa Haapasalo, Reetta Kälviäinen, Kasper Katisko, Johanna Krüger, Eino Solje

**Affiliations:** 1 Institute of Clinical Medicine–Neurology, University of Eastern Finland, Kuopio, Finland; 2 Neuro Center–Neurology, Kuopio University Hospital, Kuopio, Finland; 3 Law School, University of Eastern Finland, Joensuu, Finland; 4 Research Unit of Clinical Medicine, Department of Neurology, University of Oulu, Oulu, Finland; 5 Medical Research Center, Oulu University Hospital, Oulu, Finland; 6 Neurocenter, Neurology, Oulu University Hospital, Oulu, Finland; 7 A. I. Virtanen Institute for Molecular Sciences, University of Eastern Finland, Kuopio, Finland

## Abstract

**Importance:**

Previous studies have described a potential association between epilepsy and frontotemporal dementia (FTD), but no systematic data are available.

**Objective:**

To determine whether epilepsy is more prevalent in patients with FTD than in healthy controls (HCs) or patients with Alzheimer disease (AD).

**Design, Setting, and Participants:**

In this case-control study, we compared the prevalence of epilepsy and purchases of antiseizure medicines (ASMs) among patients with FTD, matched HCs, and patients with AD from 2 early-onset dementia diagnostics centers in the same geographic regions of Finland. AD or FTD diagnoses were made between January 1, 2010, and December 31, 2021. Data were analyzed from January 26, 2024, to January 16, 2025.

**Main Outcomes and Measures:**

The primary outcome was to describe the prevalence of epilepsy in patients with FTD, covering the time period from 10 years before to 5 years after the FTD diagnosis. We used *International Statistical Classification of Diseases, Tenth Revision* codes to identify all patients with epilepsy and tracked purchases of ASMs.

**Results:**

The study cohort included 245 patients with FTD (121 female [49.4%], 124 male [50.6%]; mean [SD] age, 65.2 [8.7] years), 2416 matched HCs (1190 female [49.3%], 1226 male [50.7%]; mean [SD] age, 65.0 [8.5] years), and 1326 patients with AD (777 female [58.6%], 549 male [41.4%]; mean [SD] age, 71.7 [9.8] years). The prevalence of epilepsy was higher in the FTD group compared with the HC group (3.3% vs 0.8%, respectively; *P* = .002) and AD group (3.3% vs 1.4%, respectively; *P* = .01) 10 years before FTD diagnosis. At the year of the diagnosis, the prevalence was 6.5% in patients with FTD, 1.8% in HCs (FTD vs HC difference, 4.7 percentage points [ppt] [95% CI, 2.2-8.6 ppt]; *P* < .001), and 5.0% in patients with AD (FTD vs AD difference, 1.6 ppt [95% CI, −1.2 to 5.5 ppt]; *P* = .32); at 5 years after the diagnosis, the prevalence was 11.2% in patients with FTD, 2.2% in HCs (FTD vs HC difference, 9.0 ppt [95% CI, 5.0-14.6 ppt]; *P* < .001), and 6.9% in patients with AD (FTD vs AD difference, 4.2 ppt [95% CI, 0-10.0 ppt]; *P* = .05). ASM purchases were made significantly more often among patients with FTD (10.2%) compared with HCs (1.8%) and patients with AD (4.2%) (FTD vs HC difference, 8.4 ppt [95% CI, 5.2-12.9 ppt]; *P* < .001; FTD vs AD difference, 6.1 ppt [95% CI, 2.6-10.6 ppt]; *P* < .001) at all time points and increased during the study period.

**Conclusions and Relevance:**

This case-control study found a higher prevalence of epilepsy and increased ASM use among patients with FTD compared with HCs and patients with AD , suggesting an association between epileptic abnormalities and the pathophysiology of FTD. Further studies are warranted to investigate a potential overlap in the pathophysiologic mechanisms of epilepsy and FTD.

## Key Points

**Question:**

Is there a significant co-occurrence of epilepsy and frontotemporal dementia (FTD)?

**Findings:**

This case-control study including 245 patients found a high prevalence of epilepsy as a comorbidity of FTD among patients who had epilepsy several years before their FTD diagnosis. Antiseizure medication purchases were significantly more common among patients with FTD compared with healthy controls and patients with Alzheimer disease.

**Meaning:**

Co-occurring epilepsy exacerbates the disease burden in patients with neurodegenerative diseases; enhancing knowledge of the comorbidity between epilepsy and FTD could lead to more precise and comprehensive diagnostics and treatment of these conditions as well as new pathophysiologic findings.

## Teaser

This case-control study assesses the prevalence of epilepsy as a comorbidity of frontotemporal dementia (FTD) as well as the frequency of antiseizure medication purchases among patients with FTD compared with health controls and patients with Alzheimer disease.

## Introduction

Frontotemporal dementia (FTD) comprises a group of neurodegenerative diseases with a wide spectrum of clinical phenotypes.^1,2,3^ It is the most common neurodegenerative disease among younger patients but is also acknowledged as the third most common neurodegenerative disease across all age groups.^4^ The phenotypes of FTD can vary from behavioral to linguistic presentations as well as to involvement of motor symptoms.^5^ The most common genetic cause of FTD is the hexanucleotide repeat expansion in the chromosome 9 open reading frame 72 gene (*C9orf72*).^2,6^ *C9orf72* repeat expansion can be found in approximately 25% of familial FTD cases and also in a smaller proportion of sporadic cases. It is especially prevalent in Scandinavia^1,7^ and Finland.^6,8,9^

Epilepsy is a frequent comorbidity of neurodegenerative diseases, and its connection to Alzheimer disease (AD) is already well established. Neurodegenerative diseases are a major etiology for epilepsy in elderly people.^10^ The prevalence of epilepsy in patients with other neurodegenerative diseases, including FTD, is less clearly defined. Some reports suggest that the incidence of seizures is lower in other neurodegenerative diseases than in AD.^10,11^ However, there is some evidence suggesting a higher risk for epilepsy in the elderly population with FTD compared with age-matched controls, but to our knowledge, these results have not been investigated in larger cohorts.^10,11^

The seizures reported in patients with any neurodegenerative disease have mostly been focal onset seizures,^10,12^ although the seizure semiology is not specific to neurodegenerative diseases.^10,12^ Some reports have indicated a higher risk of dementia-related epilepsy in early-onset patients^10,12^ and suggest that seizures could potentially be the first manifestation of the neurodegenerative process.^10,12^ To our knowledge, no systematic data on the co-occurrence of FTD and epilepsy exist.

## Methods

This case-control study was part of clinical trial NCT06209515,^13^ which is being coordinated by Neurocenter Finland, and was approved by the Finnish Social and Health Data Permit Authority Findata. According to Finnish legislation, informed consent is not required for retrospective studies if the study patients have not been contacted. The study followed the Strengthening the Reporting of Observational Studies in Epidemiology (STROBE) reporting guideline.

Data were collected from patients residing in the provinces of Northern Ostrobothnia and Northern Savonia, Finland. All patients in these provinces with a suspected neurodegenerative disease onset before age 65 years are referred to university hospitals, namely Kuopio University Hospital and Oulu University Hospital. The data were collected between January 1, 2010, and December 31, 2021, from patient registries. To confirm the specificity of the data, all diagnoses of neurodegenerative diseases (N = 12 490) were further validated via a manual review of patient charts (conducted by K.A., S.H., A. Kivisild, A.L., I.R., H.S., L.T., P.H., K.K., J.K, and E.S.) and health care records according to the relevant diagnostic criteria.^14,15,16^

We included approximately 10 healthy control (HC) individuals for each patient with FTD; these HCs were randomly selected from corresponding hospitals by Statistics Finland^17^ and were matched according to age, sex, and geographic location. As a second reference group, we recruited patients with confirmed AD.

Clinical data were retrieved from the corresponding hospital data lakes and forwarded to the Finnish Social and Health Data Permit Authority and Statistics Finland for merging with national registers. (Finland’s national social security number system makes it possible to retrieve data from each individual living in Finland from the national registers.) Data regarding antiseizure medicine (ASM) purchases were collected from the national reimbursement register of the Finnish Social Insurance Institution by searching for all ASMs under the Anatomical Therapeutic Chemical code group N03. From these data, we evaluated whether patients had bought any ASMs during the study period at cross-sectional time points and cumulatively. Prevalence of epilepsy was evaluated by searching for *International Statistical Classification of Diseases, Tenth Revision* (*ICD-10*) codes G40* from Care Register for Health Care, Hilmo, which is maintained by the Finnish Institute for Health and Welfare and includes information on the number of patients, *ICD-10* diagnoses, and treatment periods in outpatient clinics and hospital wards for both specialized and basic health care. In Finland, the diagnosis of epilepsy is always made by a neurologist.

### Statistical Analysis

Because the examined variables were mainly binary, the differences between the 3 study groups were assessed using 2-sided Pearson χ^2^ tests; statistical significance was established at *P* < .05. FTD was used as the reference group, and differences with the HC and AD groups were tested separately. We also examined the differences in age-stratified blocks, which is especially important for the comparison of the FTD and AD groups given that AD patients are older on average. As a robustness check, we examined differences between the FTD and HC groups using conditional logit models, which is the preferred method for matched case-control data with variable matching ratios. In these models, each case is only compared with each individual’s matched control.

Data were analyzed from January 26, 2024, to January 16, 2025. All analyses were conducted using Stata, version 17 (StataCorp LLC).

## Results

The study cohort included 245 patients with FTD (121 female [49.4%], 124 male [50.6%]; mean [SD] age, 65.2 [8.7] years), 2416 matched HCs (1190 female [49.3%], 1226 male [50.7%]; mean [SD] age, 65.0 [8.5] years), and 1326 patients with AD (777 female [58.6%], 549 male [41.4%]; mean [SD] age, 71.7 [9.8] years). Due to the matched age and sex, there were no differences in their distribution between the FTD and HC groups, whereas patients with AD were slightly older and more often female. Detailed study cohort characteristics are provided in the Table.

**Table.** Patient Characteristics

Characteristic	Patients with FTD (n = 245)	Patients with AD (n = 1326)	HCs (n = 2416)	*P* value	
				FTD vs AD	FTD vs HC
Age at diagnosis, mean (SD), y	65.2 (8.7)	71.7 (9.8)	65.0 (8.5)	<.001	.72
Sex, No. (%)					
Female	121 (49.4)	777 (58.6)	1190 (49.3)	.007	.98
Male	124 (50.6)	549 (41.4)	1226 (50.7)	.10	.99

Abbreviations: AD, Alzheimer disease; HC, healthy control; FTD, frontotemporal dementia.

**Figure 1.**
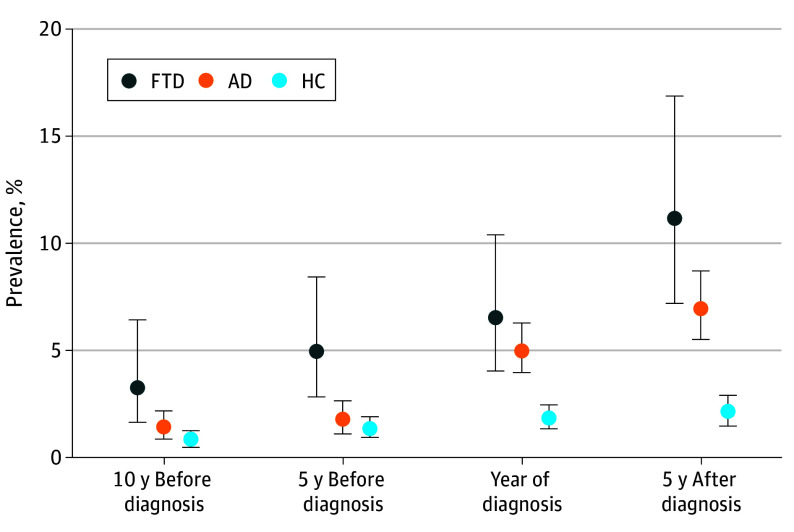
Prevalence of Epilepsy Among the Study Groups at Different Time Points Error bars represent 95% CIs. AD indicates Alzheimer disease; HC, healthy control; FTD, frontotemporal dementia.

Any *ICD-10* diagnosis code in the group G40* was used as an indicator of having epilepsy. We examined the prevalence of epilepsy at 4 separate time points: 10 and 5 years prior to FTD diagnosis, the year of diagnosis, and 5 years after diagnosis. Ten years prior to FTD diagnosis, the prevalence of epilepsy was 3.3% (95% CI, 1.6%-6.4%) in the FTD group, 0.8% (95% CI, 0.5%-1.2%) in the HC group, and 1.4% (95% CI, 0.9%-2.1%) in the AD group; the difference was statistically significant between the FTD and HC groups (2.5 percentage points [ppt] [95% CI, 0.8-5.5 ppt]; *P* < .001) and between the FTD and AD groups (1.9 ppt [95% CI, 0.1-5.0 ppt]; *P* = .03). At 5 years before FTD diagnosis, epilepsy was detected in 4.9% (95% CI, 2.8%-8.4%) of patients with FTD, 1.3% (95% CI, 0.9%-1.9%) of HCs, and 1.7% (95% CI, 1.2%-2.6%) of patients with AD; the difference was statistically significant between the FTD and HC groups (3.6 ppt [95% CI, 1.4-7.1 ppt]; *P* < .001) and between the FTD and AD groups (3.2 ppt [95% CI, 0.9-6.7 ppt]; *P* = .002). At the year of FTD diagnosis, the prevalence of epilepsy was 6.5% (95% CI, 4.0%-10.4%) in the FTD group, 1.8% (95% CI, 1.4%-2.4%) in the HC group, and 5.0% (95% CI, 3.9%-6.3%) in the AD group; the difference between the FTD and HC groups was statistically significant (4.7 ppt [95% CI, 2.2-8.6 ppt]; *P* < .001), but the difference between the FTD and AD groups was not (1.6 ppt [95% CI, −1.2 to 5.5 ppt]; *P* = .32). Five years after FTD diagnosis, the prevalence of epilepsy was 11.2% (95% CI, 7.2%-16.9%) in the FTD group, 2.2% (95% CI, 1.7%-2.9%) in the HC group (FTD vs HC difference, 9.0 ppt [95% CI, 5.0-14.6 ppt]; *P* < .001), and 6.9% (95% CI, 5.5%-8.7%) in the AD group (FTD vs AD difference, 4.2 ppt [95% CI, 0-10.0 ppt]; *P* = .05) among those still alive. The prevalence of epilepsy in FTD, AD, and HC subjects at different cross-sectional time points is presented in Figure 1.

We also compared the prevalence of epilepsy between the groups separately in male and female patients on the year of diagnosis. Among female patients, the prevalence of epilepsy was 7.4% in the FTD group at the year of diagnosis, while it was 1.1% in the HC group (FTD vs HC difference, 6.3 ppt [95% CI, 2.8-12.5 ppt]; *P* < .001) and 4.4% in the AD group (FTD vs AD difference, 3.1 ppt [95% CI, −0.8 to 9.3 ppt]; *P* = .14). Among male patients, the prevalence of epilepsy was 5.7% in the FTD group, 2.5% in the HC group (FTD vs HC difference, 3.1 ppt [95% CI, 0-8.7 ppt]; *P* = .005), and 5.8% in the AD group (FTD vs AD difference, −0.2 ppt [95% CI, −3.9 to 5.6 ppt]; *P* = .94).

Furthermore, we evaluated epilepsy prevalence in 6 different age groups (30-39 years, 40-49 years, 50-59 years, 60-69 years, 70-79 years, and 80 years and older) at the year of diagnosis. Patients in the age groups of 30-49 years and 80 years and older were excluded due to small sample sizes. In the age group of 50 to 59 years, the prevalence of epilepsy was 7.6% in patients with FTD, 1.5% in HCs (FTD vs HC difference, 6.0 ppt [95% CI, 1.2-16.4 ppt]; *P* = .003), and 3.6% in patients with AD (FTD vs AD difference, 4.0 ppt [95% CI, −2.5 to 14.5 ppt]; *P* = .25). In the age group of 60-69 years, the prevalence was 6.4% in patients with FTD, 1.8% in HCs (FTD vs HC difference, 4.6 ppt [95% CI, 1.2-10.0 ppt]; *P* = .002), and 5.2% in patients with AD (FTD vs AD difference, 1.5 ppt [95% CI, −2.5 to 8.0 ppt]; *P* = .63), and in the group of 70-79 years, it was 6.7% in FTD, 1.9% in HC (FTD vs HC difference, 4.8 ppt [95% CI, 0.5-14.1 ppt]; *P* = .02), and 4.9% in AD (FTD vs AD difference, 1.8 ppt [95% CI, −2.9 to 11.2 ppt]; *P* = .56).

**Figure 2.**
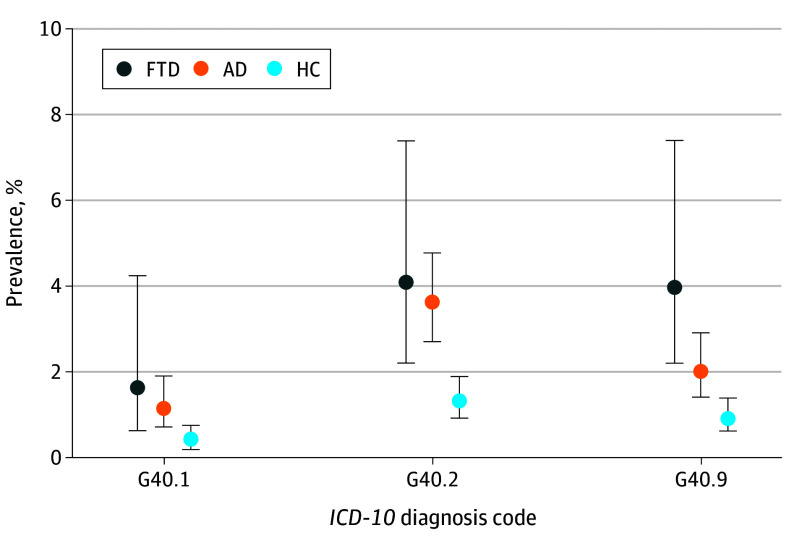
Prevalence of the Most Common Epilepsy Diagnosis Codes Diagnosis codes are from the *International Statistical Classification of Diseases, Tenth Revision* (*ICD-10*). G40.1 = Localization-related (focal) (partial) symptomatic epilepsy and epileptic syndromes with simple partial seizures. G40.2 = Localization-related (focal) (partial) symptomatic epilepsy and epileptic syndromes with complex partial seizures. G40.9 = Epilepsy, unspecified, not intractable. Error bars represent 95% CIs. AD indicates Alzheimer disease; HC, healthy control; FTD, frontotemporal dementia.

Examination of *ICD-10* codes revealed that the most common *ICD-10* codes in the FTD group were G40.2, G40.1, and G40.9, indicating that the epilepsy type either was focal onset epilepsy or could not be further defined. Detailed *ICD-10* code results are shown in Figure 2.

Next, we evaluated cumulative purchases of any ASM at the time points of 10 and 5 years prior to FTD diagnosis, at the year of the diagnosis, and 5 years after diagnosis. Ten years prior to diagnosis, the prevalence of ASM usage was 11.4% (95% CI, 8.0%-16.1%) in the FTD group, 5.0% (95% CI, 4.2%-5.9%) in the HC group, and 5.6% (95% CI, 4.4%-6.9%) in the AD group (FTD vs HC difference, 6.5 ppt [95% CI, 2.9-11.1 ppt]; *P* < .001; FTD vs AD difference, 5.8 ppt [95% CI, 2.2-10.6 ppt]; *P* < .001). At 5 years before diagnosis, the prevalence of ASM usage was 16.7% (95% CI, 12.6%-21.9%) in the FTD group, 9.1% (95% CI, 8.0%-10.3%) in the HC group, and 10.3% (95% CI, 8.8%-12.1%) in the AD group (FTD vs HC difference, 7.6 ppt [95% CI, 3.3-12.9 ppt]; *P* < .001; FTD vs AD difference, 6.4 ppt [95% CI, 1.9-11.8 ppt]; *P* = .004). At the year of FTD diagnosis, the prevalence of ASM usage was 28.6% (95% CI, 23.3%-34.5%) in the FTD group, 14.6% (95% CI, 13.2%-16.1%) in the HC group, and 17.8% (95% CI, 15.8%-20.0%) in the AD group (FTD vs HC difference, 14.0 ppt [95% CI, 8.5-20.1 ppt]; *P* < .001; FTD vs AD difference, 10.8 ppt [95% CI, 5.1-17.0 ppt]; *P* < .001). Five years after FTD diagnosis, the prevalence of ASM usage was 40.0% (95% CI, 32.9%-47.5%) in the FTD group, 18.8% (95% CI, 17.2%-20.5%) in the HC group, and 23.8% (95% CI, 20.6%-26.0%) in the AD group (FTD vs HC difference, 21.2 ppt [95% CI, 13.9-28.9 ppt]; *P* < .001; FTD vs AD difference, 16.8 ppt [95% CI, 9.2-24.8 ppt]; *P* < .001) among patients who had ever purchased ASMs. We tested the accuracy of epilepsy diagnoses further by evaluating the overlap between diagnostic codes of epilepsy and ASM claims and found that over 90% of those having a G40* diagnosis also made ASM purchases.

**Figure 3.**
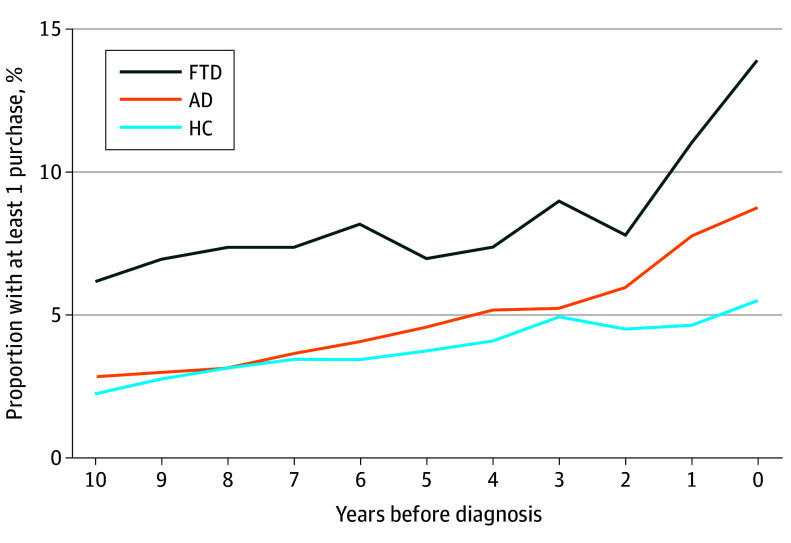
Prevalence of Antiseizure Medicine Purchases Before FTD Diagnosis Error bars represent 95% CIs. AD indicates Alzheimer disease; HC, healthy control; FTD, frontotemporal dementia.

Valproic acid was the most purchased ASM, and it was purchased significantly more often in the FTD group (10.2%) compared with the HC group (1.8%) and AD group (4.2%) (FTD vs HC difference, 8.4 ppt [95% CI, 5.2-12.9 ppt]; *P* < .001; FTD vs AD difference, 6.1 ppt [95% CI, 2.6-10.6 ppt]; *P* < .001). After valproic acid, the most frequently purchased ASMs were pregabalin, gabapentin, clonazepam, and carbamazepine. To further increase the specificity to find ASMs used only for epilepsy, we excluded pregabalin, gabapentin, valproic acid, clonazepam and lamotrigine, all of which may have other indications for use. After making these exclusions, the prevalence of ASM use was 5.3%-7.0% in the FTD group, 2.1%-3.4% in the HC group, and 2.1%-5.1% in the AD group (from 10 years before to the year of diagnosis, respectively). The differences between groups were statistically significant at all time points except between the FTD and AD groups at the year of FTD diagnosis. Purchases of any ASM before the diagnosis of FTD or AD compared with HC purchases are presented in Figure 3.

Finally, we investigated the rate of mortality in patients with FTD with and without epilepsy. In the FTD group without confounder adjustments, the mortality rate was higher among patients without epilepsy (47.6%) than among those with epilepsy (32.4%). However, after adjusting for age at the time of FTD diagnosis, the risk of death was not significant between patients with or without epilepsy.

## Discussion

In this study, we found a higher prevalence of epilepsy among patients with FTD compared with HCs or patients with AD 10 years before FTD diagnosis. This finding remained statistically significant at all study time points. The association between FTD and epilepsy is further supported by the fact that ASM purchases were significantly more common among patients with FTD during the prediagnostic period. Moreover, there was a notable further increase in the prevalence of ASM purchases approximately 2 years before the diagnosis. Our findings also suggest that increased mortality was not associated with comorbidity of epilepsy among patients with FTD. The cumulative risk of epilepsy until 5 years after the diagnosis was approximately 11% in patients with FTD.

Previous studies have proposed that epilepsy is a more common comorbidity of AD than of other neurodegenerative diseases.^10,11^ In the present study, the results indicated the opposite, as the prevalence of epilepsy was similar or higher among patients with FTD compared with patients with AD. Previous studies have reported that the prevalence of epilepsy among patients with FTD varies between 2.6% and 7.1%,^7,10^ whereas the prevalence in our dataset was approximately 11%. The prevalence of epilepsy has been reported to be 0.7% in healthy adults aged 55 to 65 years and 1.2% in adults aged 85 to 94 years.^18^ There was a similar pattern in our findings, which show that the prevalence of epilepsy increased with age. We found that the overall prevalence of epilepsy appeared to be higher even in HCs compared with the prevalence of 0.8% to 0.9% reported previously.^18^ In particular, the focal onset seizures can be very subtle and therefore very hard to recognize, which might lead to an underestimation of the actual occurrence of seizures.^18,19^ Moreover, the whole clinical spectrum of symptoms in all neurodegenerative diseases, and especially in FTD, can be so diverse that focal seizures without any motor symptoms remain unrecognized.^19^ The symptoms of seizures can be very similar to the symptoms of FTD itself, including altered responsiveness, arrest in speech or behavior, oral automatisms, and focal motor phenomena. In adult-onset epilepsy, there might be a 10% copathology with neurodegenerative disease,^11^ but in general, the etiology of epilepsy might be classified as unknown until the symptoms of a neurodegenerative disorder begin, thus revealing the etiology.^11^ This indicates that the proposed connection between epilepsy and FTD might be even stronger than is known thus far.

Life expectancy after FTD diagnosis varies from 3 to 14 years.^2,20^ To our knowledge, there are no systematic data available about FTD and comorbid epilepsy, thus the effects of epilepsy on the survival of patients with FTD have not been previously assessed. Our study assessed the relationship between epilepsy and mortality in patients with FTD, and the results indicate that epilepsy as a comorbidity did not lead to increased mortality among patients with FTD.

In patients with FTD, neuropsychiatric and behavioral symptoms are commonly manifested even years before the actual diagnosis.^21,22,23^ Consequently, the patients may first receive a psychiatric diagnosis and the requisite treatments. Active use of psychopharmacologic medications is more common among patients with FTD than for other neurodegenerative diseases, such as AD.^21,22,23,24^ Several of these medications—including valproic acid, lamotrigine, and benzodiazepines—are used as off-label drugs to alleviate behavioral symptoms.^21,22,23,25^ Gabapentinoids (pregabalin, gabapentin) form a group of medications that can be used as ASMs but also to treat neuropathic pain^26^ and anxiety.^27^ In the present study, we found that a higher proportion of individuals in all 3 groups had bought ASMs during the years before the year of diagnosis relative to actual epilepsy diagnoses. The underlying reason for this discrepancy could be that the ASMs may have been used to treat psychiatric or behavioral symptoms or neuropathic pain in these individuals.

Previous studies have proposed that disturbances in the different neurotransmitter systems are associated with neuronal degeneration and progressive atrophy in the brains of patients with FTD.^10,28^ Similar changes may also be linked to epileptogenesis.^10^ The fact that epilepsy may begin years before the clinical diagnosis of a neurodegenerative disease is evident in our data. This result suggests that specific pathophysiologic processes other than neurodegeneration might underlie epileptogenesis. Increased risk of seizures is often connected to neuropathologic changes, but there might be epileptogenic comorbidity between neurodegenerative diseases and epilepsy,^10,11^ perhaps at least partially related to changes in the neurotransmitter systems involving glutamate, acetylcholine, or γ-aminobutyric acid. Alterations in these systems play an important role in epileptogenesis, and dysregulation of the balance between neuronal excitation and inhibition may also contribute to the early pathologic changes in the degenerating brain.^29,30,31,32^ Changes in functional connectivity networks have been reported to be associated with FTD symptoms even before brain atrophy is apparent.^33^

In FTD, neuronal loss occurs especially in the frontal, anterior, and temporal lobes, but there can also be changes in the hippocampal area, which is an important structure in the pathogenesis of epilepsy.^34^ The current data collectively suggest that there are possibly similar alterations in the neurotransmitter systems, which could lead to dysregulation of neuronal excitation and inhibition. In both, functional connectivity networks as well as brain structures may underlie the pathogenesis of both FTD and epilepsy, suggesting that there may be overlap in the pathophysiologic mechanisms of these 2 disorders.

### Strengths and Limitations

The major strengths of our study include the comprehensive population-based data collection from 2 university hospitals—both of which are highly specialized in epilepsy and neurodegenerative diseases—in 2 specified geographic regions with minimal selection bias. These university hospitals serve as secondary centers where all patients with suspected early-onset dementias are referred. In the Finnish epilepsy care pathway, these centers are secondary and tertiary clinics and, as members of the European Reference Network EpiCARE, participate in an international consulting network. Data collection over a 12-year period minimized the impact of annual variability in the results, which enhances the reliability of our findings. All the FTD and AD diagnoses were manually confirmed using the current diagnostic criteria of each disease. Due to our systematic methodology in data collection, high specificity and sensitivity in detecting patients with neurodegenerative diseases are reached. The diagnostic path of early-onset dementia constitutes a high-quality multidisciplinary assessment using structural and functional brain imaging and other biomarker studies (CSF biomarkers, genetic testing) and comprehensive neuropsychologic assessments.

This study also has some limitations. Even though the data collection of patients with neurodegenerative disease was done very meticulously, the epilepsy types could only be defined at the level of the current *ICD-10* codes, which does not reflect the epilepsy phenotypes accurately. However, the epilepsy diagnoses were searched using *ICD-10* codes, which can be considered very reliable in Finland because the diagnosis of epilepsy is always made by neurologists and the Finnish health care register system mandates *ICD-10* coding for every visit. Given the health care structure in Finland, it is extremely likely that an individual fulfilling the criteria of epilepsy would be recorded in our registers and thus identified in our study. Additionally, stricter requirements for multiple separate G40* visits for each patient did not significantly change the data. The diagnosis code R56* for convulsions was excluded, meaning that the present data exclude patients who had a single seizure without a diagnosis of epilepsy. An additional limitation is that our data are derived from only 2 major provinces in Finland. Despite this limitation, the study population represented 6 467 028 person-years, making the statistical power in the analyses robust. Because our registers were available beginning in 1998, it is possible that some epilepsy diagnoses were recorded only before 1998 and therefore not identified. However, because a diagnosis of epilepsy is chronic, and because follow-up is always provided in Finland, missing data due to this scenario is unlikely.

## Conclusions

To date, and to our knowledge, this case-control study represents the largest and most comprehensive analysis of epilepsy as a comorbidity of FTD. We found a significantly higher prevalence of epilepsy diagnoses and use of ASMs among patients with FTD compared with a cognitively healthy control population and patients with AD. The risk of epilepsy increased several years before the clinical onset of FTD symptoms, which could be due to overlapping neuropathologic and neurophysiologic changes between epilepsy and early FTD. The observed association between FTD and epilepsy was not limited to any specific seizure type, and comorbid epilepsy did not increase mortality among the patients with FTD.

## Notes

Supplement.
Data Sharing Statement

## References

[1] Bang J, Spina S, Miller BL. Frontotemporal dementia. Lancet. 2015;386(10004):1672-1682. doi:10.1016/S0140-6736(15)00461-4 26595641 PMC5970949

[2] Majounie E, Renton AE, Mok K, ; Chromosome 9-ALS/FTD Consortium; French Research Network on FTLD/FTLD/ALS; ITALSGEN Consortium. Frequency of the C9orf72 hexanucleotide repeat expansion in patients with amyotrophic lateral sclerosis and frontotemporal dementia: a cross-sectional study. Lancet Neurol. 2012;11(4):323-330. doi:10.1016/S1474-4422(12)70043-1 22406228 PMC3322422

[3] Olney NT, Spina S, Miller BL. Frontotemporal dementia. Neurol Clin. 2017;35(2):339-374. doi:10.1016/j.ncl.2017.01.008 28410663 PMC5472209

[4] Antonioni A, Raho EM, Lopriore P, . Frontotemporal dementia, where do we stand? a narrative review. Int J Mol Sci. 2023;24(14):11732. doi:10.3390/ijms241411732 37511491 PMC10380352

[5] Seltman RE, Matthews BR. Frontotemporal lobar degeneration: epidemiology, pathology, diagnosis and management. CNS Drugs. 2012;26(10):841-870. doi:10.2165/11640070-000000000-00000 22950490

[6] Kaivorinne AL, Bode MK, Paavola L, . Clinical characteristics of C9ORF72-linked frontotemporal lobar degeneration. Dement Geriatr Cogn Dis Extra. 2013;3(1):251-262. doi:10.1159/000351859 24052799 PMC3776392

[7] Muroni A, Floris G, Polizzi L, . Does epilepsy contribute to the clinical phenotype of C9orf72 mutation in fronto-temporal dementia? Epilepsy Behav. 2022;133:108783. doi:10.1016/j.yebeh.2022.108783 35752055

[8] Logroscino G, Piccininni M, Graff C, ; FRONTIERS group. Incidence of syndromes associated with frontotemporal lobar degeneration in 9 European countries. JAMA Neurol. 2023;80(3):279-286. doi:10.1001/jamaneurol.2022.5128 36716024 PMC9887528

[9] Rostalski H, Korhonen V, Kuulasmaa T, . A novel genetic marker for the C9orf72 repeat expansion in the Finnish population. J Alzheimers Dis. 2021;83(3):1325-1332. doi:10.3233/JAD-210599 34397416

[10] Beagle AJ, Darwish SM, Ranasinghe KG, La AL, Karageorgiou E, Vossel KA. Relative incidence of seizures and myoclonus in Alzheimer’s disease, dementia with Lewy bodies, and frontotemporal dementia. J Alzheimers Dis. 2017;60(1):211-223. doi:10.3233/JAD-170031 28826176 PMC5608587

[11] Neri S, Mastroianni G, Gardella E, Aguglia U, Rubboli G. Epilepsy in neurodegenerative diseases. Epileptic Disord. 2022;24(2):249-273. doi:10.1684/epd.2021.1406 35596580

[12] Wang X, Loi SM, Foster E, Chen Z, Velakoulis D, Kwan P. Predictors of new-onset epilepsy in people with younger-onset neurocognitive disorders. Front Aging Neurosci. 2021;13:637260. doi:10.3389/fnagi.2021.637260 33815091 PMC8010684

[13] Sociodemographic factors and criminal behaviour preceding neurodegenerative disease—retrospective register study (DEGERWD). ClinicalTrials.gov identifier: NCT06209515. Updated January 31, 2024. Accessed April 21, 2025. https://www.clinicaltrials.gov/search?term=NCT06209515

[14] Rascovsky K, Hodges JR, Knopman D, . Sensitivity of revised diagnostic criteria for the behavioural variant of frontotemporal dementia. Brain. 2011;134(pt 9):2456-2477. doi:10.1093/brain/awr179 21810890 PMC3170532

[15] Gorno-Tempini ML, Hillis AE, Weintraub S, . Classification of primary progressive aphasia and its variants. Neurology. 2011;76(11):1006-1014. doi:10.1212/WNL.0b013e31821103e6 21325651 PMC3059138

[16] McKhann GM, Knopman DS, Chertkow H, . The diagnosis of dementia due to Alzheimer’s disease: recommendations from the National Institute on Aging-Alzheimer’s Association workgroups on diagnostic guidelines for Alzheimer’s disease. Alzheimers Dement. 2011;7(3):263-269. doi:10.1016/j.jalz.2011.03.005 21514250 PMC3312024

[17] Statistics Finland. Accessed April 21, 2025. https://stat.fi/en

[18] de la Court A, Breteler MM, Meinardi H, Hauser WA, Hofman A. Prevalence of epilepsy in the elderly: the Rotterdam Study. Epilepsia. 1996;37(2):141-147. doi:10.1111/j.1528-1157.1996.tb00005.x 8635424

[19] Baker J, Libretto T, Henley W, Zeman A. The prevalence and clinical features of epileptic seizures in a memory clinic population. Seizure. 2019;71:83-92. doi:10.1016/j.seizure.2019.06.016 31226566

[20] Kansal K, Mareddy M, Sloane KL, . Survival in frontotemporal dementia phenotypes: a meta-analysis. Dement Geriatr Cogn Disord. 2016;41(1-2):109-122. doi:10.1159/000443205 26854827

[21] Bialer M. Why are antiepileptic drugs used for nonepileptic conditions? Epilepsia. 2012;53(suppl 7):26-33. doi:10.1111/j.1528-1167.2012.03712.x 23153207

[22] Poetter CE, Stewart JT. Treatment of indiscriminate, inappropriate sexual behavior in frontotemporal dementia with carbamazepine. J Clin Psychopharmacol. 2012;32(1):137-138. doi:10.1097/JCP.0b013e31823f91b9 22217950

[23] Tsai RM, Boxer AL. Treatment of frontotemporal dementia. Curr Treat Options Neurol. 2014;16(11):319. doi:10.1007/s11940-014-0319-0 25238733 PMC4920050

[24] Chow TW, Mendez MF. Goals in symptomatic pharmacologic management of frontotemporal lobar degeneration. Am J Alzheimers Dis Other Demen. 2002;17(5):267-272. doi:10.1177/153331750201700504 12392261 PMC5841918

[25] Katisko K, Krüger J, Soppela H, . Psychopharmacological medication use in frontotemporal dementia at the time of diagnosis: comparison with Alzheimer’s disease. J Alzheimers Dis. 2023;95(2):677-685. doi:10.3233/JAD-230494 37574738

[26] Wiffen PJ, Derry S, Bell RF, . Gabapentin for chronic neuropathic pain in adults. Cochrane Database Syst Rev. 2017;6(6):CD007938. doi:10.1002/14651858.CD007938.pub4 28597471 PMC6452908

[27] Supasitthumrong T, Bolea-Alamanac BM, Asmer S, Woo VL, Abdool PS, Davies SJC. Gabapentin and pregabalin to treat aggressivity in dementia: a systematic review and illustrative case report. Br J Clin Pharmacol. 2019;85(4):690-703. doi:10.1111/bcp.13844 30575088 PMC6422659

[28] Murley AG, Rowe JB. Neurotransmitter deficits from frontotemporal lobar degeneration. Brain. 2018;141(5):1263-1285. doi:10.1093/brain/awx327 29373632 PMC5917782

[29] Akyuz E, Polat AK, Eroglu E, Kullu I, Angelopoulou E, Paudel YN. Revisiting the role of neurotransmitters in epilepsy: an updated review. Life Sci. 2021;265:118826. doi:10.1016/j.lfs.2020.118826 33259863

[30] Huber N, Korhonen S, Hoffmann D, . Deficient neurotransmitter systems and synaptic function in frontotemporal lobar degeneration—insights into disease mechanisms and current therapeutic approaches. Mol Psychiatry. 2022;27(3):1300-1309. doi:10.1038/s41380-021-01384-8 34799692 PMC9095474

[31] Ferrer I. Neurons and their dendrites in frontotemporal dementia. Dement Geriatr Cogn Disord. 1999;10(suppl 1):55-60. doi:10.1159/000051214 10436342

[32] Vossel KA, Tartaglia MC, Nygaard HB, Zeman AZ, Miller BL. Epileptic activity in Alzheimer’s disease: causes and clinical relevance. Lancet Neurol. 2017;16(4):311-322. doi:10.1016/S1474-4422(17)30044-3 28327340 PMC5973551

[33] Dopper EG, Rombouts SA, Jiskoot LC, . Structural and functional brain connectivity in presymptomatic familial frontotemporal dementia. Neurology. 2014;83(2):e19-e26. doi:10.1212/WNL.0000000000000583 25002573

[34] Wang J, Wang B, Zhou T. The advance on frontotemporal dementia (FTD)'s neuropathology and molecular genetics. Mediators Inflamm. 2022;2022:5003902. doi:10.1155/2022/5003902 36274975 PMC9584734

