# Supplementary material for: Prevalence of Epilepsy in Frontotemporal Dementia and Timing of Dementia Diagnosis
Source: JAMA Neurol. 2025 Jun 2;82(7):715–21. doi: 10.1001/jamaneurol.2025.1358 (PMC12131175; doi:10.1001/jamaneurol.2025.1358)
Supplement: Supplement. — Data Sharing Statement [file jamaneurol-e251358-s001.pdf]

# Data Sharing Statement

Kilpeläinen. Prevalence of Epilepsy in Frontotemporal Dementia and Timing of Dementia Diagnosis. *JAMA Neurol*. Published June 02, 2025. doi:10.1001/jamaneurol.2025.1358

**Data**
**Data available:** No

**Additional Information**
**Explanation for why data not available:** According to Finnish privacy legislation individual level data can not be shared. However group level data can be shared upon reasonable request to corresponding author.
